# Adult human periodontal ligament-derived stem cells delay retinal degeneration and maintain retinal function in RCS rats

**DOI:** 10.1186/s13287-017-0731-y

**Published:** 2017-12-22

**Authors:** Li Huang, Zongyi Li, Haibin Tian, Weiguo Wang, Dawei Cui, Zhe Zhou, Xiao Chen, Herman S. Cheung, Guo-tong Xu, Yu Chen

**Affiliations:** 1 Department of Laboratory Medicine, First Affiliated Hospital, College of Medicine, Zhejiang University, Hangzhou, 310003 China; 2 Key Laboratory of Clinical In Vitro Diagnostic Techniques of Zhejiang Province, Hangzhou, 310003 China; 3 Qingdao University, Qingdao, 266071 China; 4 Department of Ophthalmology of Shanghai Tenth People’s Hospital, and Laboratory of Clinical Visual Science of Tongji Eye Institute, and Department of Pharmacology, Tongji University School of Medicine, Shanghai, 200092 China; 5 The Stem Cell Research Center and the Stem Cell Bank, Tongji University School of Medicine, Shanghai, 200092 China; 6 Department of Oral and Maxillofacial Surgery, First Affiliated Hospital, College of Medicine, Zhejiang University, Hangzhou, 310003 China; 7 Key Laboratory of Precision Diagnosis and Treatment for Hepatobiliary and Pancreatic Tumor of Zhejiang Province, Hangzhou, 310003 China; 8 Department of Biomedical Engineering, College of Engineering, University of Miami, Coral Gables, FL 33146 USA; 9 Geriatric Research, Education and Clinical Center (GRECC), Miami Veterans Affairs (VA) Medical Center, Miami, FL 33146 USA

**Keywords:** Periodontal ligament, Stem cells, Transplantation, Retinal degeneration, Therapy

## Abstract

**Background:**

Retinal degeneration (RD) is a leading cause of irreversible blindness, affecting millions of people worldwide. Stem cell transplantation has been considered a promising therapy for retinal degenerative diseases. This study aimed to investigate the therapeutic potential of human periodontal ligament-derived stem cells (hPDLSCs) for intervention in the progress of this degeneration in the Royal College Surgeons (RCS) rat.

**Methods:**

hPDLSCs were injected into the subretinal space of 3-week-old RCS rats. Control animals received a phosphate-buffered saline injection or were untreated. Retinal function was assessed by electroretinography recording. Eyes were collected afterward for histology and molecular studies.

**Results:**

Retinal function maintenance was observed at 2 weeks and persisted for up to 8 weeks following hPDLSC transplantation. Retinal structure preservation was demonstrated in hPDLSC-transplanted eyes at 4 and 8 weeks following transplantation, as reflected in the preservation of outer nuclear layer thickness and gene expression of Rho, Crx, and Opsin. The percentage of terminal deoxynucleotidyl transferase-mediated dUTP nick-end labeling-positive apoptotic photoreceptors was significantly lower in the hPDLSC-injected retinas than in those of the control groups. hPDLSCs were also found to express multiple neurotrophic factors, including vascular endothelial growth factor, bioactive basic fibroblast growth factor, brain-derived neurotrophic factor, neurotrophin-3, insulin-like growth factor 1, nerve growth factor, and glial cell line-derived neurotrophic factor.

**Conclusions:**

Our findings suggest that hPDLSC transplantation is effective in delaying photoreceptor loss and provides significant preservation of retinal function in RCS rats. This study supports further exploration of hPDLSCs for treating RD.

**Electronic supplementary material:**

The online version of this article (doi:10.1186/s13287-017-0731-y) contains supplementary material, which is available to authorized users.

## Background

The loss of photoreceptor cells and/or their supportive retinal pigmented epithelial (RPE) cells is generally regarded to be the irreversible cause of blindness in many retinal degenerative diseases, such as retinitis pigmentosa (RP) [1], age-related macular degeneration (AMD) [2], and Stargardt disease [3]. There are currently no effective treatments for a majority of these progressive diseases, except for exudative AMD. Stem cell-based therapy is an attractive approach to treat retinal degeneration with the potential to rescue or replace degenerated cells in the retina. Neural stem cells (NSCs) have been recognized for their role in retinal repair, but ethical concerns and the limited and variable cell source may preclude their routine use [4, 5]. Embryonic stem cells (ESCs) and induced pluripotent stem cells (iPSCs) have shown the greatest experimental utility and some clinical trials are already underway using human ESC and iPSC-derived RPE transplantation to prevent photoreceptor degeneration in RP, AMD, and SD (ClinicalTrials.gov). However, the long and tedious preinduction preparation is costly and may introduce a risk of contamination and errors. In addition, ethical concerns and the risk of immune rejection still hamper the use of ESCs. The continuing effort to identify new sources of stem cells for the treatment of retinal degeneration and evaluate their engraftment behavior in disease models is urgently needed.

Dental stem cells, including dental pulp stem cells (DPSCs), stem cells from human exfoliated deciduous teeth (SHED), periodontal ligament stem cells (PDLSCs), stem cells from apical papilla (SCAP), and dental follicle progenitor cells (DFPCs), are attractive cell resources and have received extensive attention for regenerative use not only in dentistry but also for the reconstruction of nondental tissues, such as bone, muscle, vascular system, and central nervous system tissues [6]. The advantages of the use of dental stem cells include their easy isolation by noninvasive routine clinical procedures, their broad differentiation potential, minimal ethical concerns, and that they may enable autologous transplantation [7]. Moreover, human dental stem cells exhibit immunosuppressive capacities [8, 9], rendering them a good source of cells for allogeneic cell transplantation. In contrast to other commonly used mesenchymal stem cell (MSC) types, such as bone marrow MSCs (BMSCs) and adipose-derived stem cells (ADSCs), dental stem cells have advantages in terms of their accessibility with minimal donor-site morbidity, a higher proliferation rate, and a more favorable neurotrophic secretome [10–12]. In particular, dental stem cells are regarded as ecto-MSCs originating from the neural crest and have thus been considered a more appropriate cell type for neuroprotective and neuroregenerative cell therapy [13].

An emerging new therapeutic theme is the alternative use of dental stem cells for the treatment of neurodegenerative conditions in the eye [13, 14]. It was reported recently that DPSCs can be induced to differentiate into a photoreceptor phenotype [15] and retinal ganglion cell (RGC)-like cells [16]. Compared with BMSCs, transplanted human DPSCs displayed a more pronounced paracrine-mediated RGC survival and neurite outgrowth in animal models of optic nerve injury [12] and glaucoma [17]. We have shown previously that hPDLSCs can differentiate into a retinal lineage exhibiting neuronal, photoreceptor [18], and RGC markers [19] in vitro. However, their therapeutic effect in vivo has not yet been confirmed. These findings provide strong justification for the continued investigation of their engraftment behavior in disease models of retinal degeneration. The purpose of this study was to investigate the efficacy of the transplantation of hPDLSCs in RCS rats, a well-established model of RD, using functional measures and morphological criteria. This study demonstrated for the first time that the subretinal injection of hPDLSCs was effective in delaying retinal degeneration and maintaining retinal function in the RCS rat model of retinal degeneration. These findings provide the first validation of the potential therapeutic utility of hPDLSCs in treating RD.

## Methods

### Human PDLSC culture

hPDLSCs were isolated from the periodontal ligament of extracted adult third molars as described previously [18]. The study was performed in accordance with the tenets of the Declaration of Helsinki, and written informed consent was obtained from the patients after receiving approval by the Ethics Committee of the First Affiliated Hospital of Zhejiang University, China. The PDLSCs were cultured in DMEM/F12 (Life Technologies) containing 10% fetal bovine serum (FBS; Hyclone Laboratories, Logan, UT, USA) and 1% penicillin/streptomycin (Life Technologies) in a humidified 5% CO_2_ incubator at 37 °C. All reagents were obtained from Invitrogen (Carlsbad, CA, USA). The medium was changed every 2–3 days, and the cells were passaged at 80% confluency. hPDLSCs pooled from three different healthy donors (one male, two female; age 18–25 years) at passage 2–3 were utilized in this study.

### Flow cytometry

The cells were washed with phosphate-buffered saline (PBS), trypsinized, and resuspended in 2% FBS in PBS. Typically, 2 × 10^5^ cells were used for antibody labeling. The cells were incubated with each conjugated antibody in the dark for 30 min on ice or an unconjugated antibody for 1 h on ice. When using unlabeled primary antibodies, after washing with PBS the cells were subsequently stained with the appropriate fluorescent-conjugated secondary antibodies for 30 min. Following incubation, the cells were rinsed with PBS and resuspended in 500 μl of PBS. All control cells were stained with the corresponding isotype controls. Analysis was performed using a FACSCalibur flow cytometer (BD Biosciences). For each sample, a minimum of 10,000 events were recorded, and the data were compiled using FlowJo software.

### Immunocytochemistry

Cells were fixed in 4% paraformaldehyde for 30 min at room temperature and incubated with primary antibodies (Additional file 1: Table S1) overnight at 4 °C. The appropriate Alexa-488 and Alexa-594-conjugated secondary antibodies were used, and the cell nuclei were counterstained with 4′,6′-diamidino-2-phenylindole (DAPI). Images were obtained using a Zeiss confocal microscope.

### Enzyme-linked immunosorbent assay

hPDLSCs were plated in a 100-mm dish and grown to 90% confluency. The cells were then cultured with serum-free medium for 48 h. The culture supernatants and cell lysates were collected. The levels of secreted and intracellular vascular endothelial growth factor (VEGF), nerve growth factor (NGF), and brain-derived neurotrophic factor (BDNF) were measured using enzyme-linked immunosorbent assay (ELISA) kits (Elabscience) for BDNF, VEGF, and NGF according to the manufacturers’ instructions.

### Induction of adipogenesis, osteogenesis, and chondrogenesis

hPDLSCs were seeded at a density of 5 × 10^4^ cells/well in six-well plates and cultured in standard medium until they reached subconfluency. The medium was then switched to Osteogenic Differentiation Medium (Invitrogen) or Adipogenic Differentiation Medium (Invitrogen) for 2 weeks. To detect lipid formation after adipocyte differentiation, the medium was removed. The cells were then fixed with paraformaldehyde for 30 min and stained with fresh Oil Red O solution (Sigma-Aldrich) for 15 min. Osteogenic differentiation was evaluated by detecting the alkaline phosphatase activity using the colorimetric assay SigmaFast BCIP/NBT (Sigma-Aldrich).

For chondrogenesis, hPDLSCs were aggregated by hanging drop culture. Five-microliter droplets of a 1 × 10^7^ cells/ml cell suspension were seeded on the lid of a six-well culture plate. The lid was inverted and placed on a plate containing PBS. The cells were maintained for 24 h in a humidified 5% CO_2_ incubator at 37 °C. The generated hPDLSC micromasses were cultured in Chondrogenesis Differentiation Medium (Invitrogen) for 2 weeks. The cells were rinsed with PBS and fixed with 4% formaldehyde solution for 30 min to prepare for Alcian Blue staining. The development of chondrogenic differentiation was determined by staining cells with a 1% Alcian Blue solution prepared in 0.1 N HCl for 30 min. Blue staining indicates the synthesis of proteoglycans by chondrocytes.

### Subretinal transplantation

All protocols and the animal procedures in this study were carried out in accordance with National Institutes of Health (NIH) Guidelines for the Care and Use of Laboratory Animals, with approval from the animal ethics committee of Tongji University (Permit Number: TJmed-010-32). RCS rats were housed and bred in the Laboratory Animal Center of Tongji University. In-vivo transplants were performed on 3-week-old RCS rats. The cells were trypsinized and suspended at 1 × 10^5^ cells/μl in PBS prior to injection. The rats were anesthetized with 2% sodium pentobarbital. A 30-gauge needle was inserted into the vitreous chamber behind the limbus to create a channel. Subsequently, a 33-gauge needle was inserted into the subretinal space of the central retina, and 2 μl of the cells was injected. Contralateral eyes received a sham injection of PBS or no treatment as controls.

### Retinal histological and immunohistochemical examinations

After the rats were sacrificed by cervical dislocation, the eyes were enucleated immediately and fixed in 4% PFA at room temperature for 30 min. The eyes were embedded in paraffin, and sections 4 μm thick were prepared and stained with hematoxylin and eosin (H&E). The thickness of the inner nuclear layer (INL) and the outer nuclear layer (ONL) was measured at each pair of nasal and temporal points at the defined distances (in millimeters) to the optic nerve head (ONH). For cryosection preparation, the eyes were infiltrated with 30% sucrose overnight at 4 °C and embedded in optimum cutting temperature (OCT) compound. Sections 8 μm thick were cut along the horizontal meridian of the eye through the ONH before immunostaining. The same immunofluorescent staining techniques and photography methods as those already described were applied in the immunohistochemistry studies. The primary antibodies used are presented in Additional file 1: Table S1.

### Gene expression analysis by reverse transcription polymerase chain reaction (RT-PCR) and quantitative RT-PCR

Total RNA from the cells was isolated, and the first-strand cDNA was synthesized according to the manufacturer’s instructions using the RNeasy Plus Mini Kit (Qiagen) and the SuperScript® III First-Strand Synthesis System (Life Technologies). PCR was performed using gene-specific primers (Additional file 2: Table S2) and Taq DNA polymerase (Takara). After amplification, the PCR products were detected by 2% agarose gel electrophoresis. Quantitative reverse transcription polymerase chain reaction (qRT-PCR) was performed using the SYBR Green PCR Kit (Qiagen). The gene expression levels were calculated using the 2^–ΔCt^ method relative to GAPDH as a reference. The fold-changes resulting from treatment were obtained by comparing the normalized expression values for each gene in the treatment and untreated groups. The primer sequences are presented in Additional file 2: Table S2.

### Electroretinography examination

ERG recordings of the rats were obtained 2,4, and 8 weeks after cell transplantation. The ERG recordings were performed with an AVES system (Kanghuaruiming Technology Co., Ltd) following procedures described previously [20]. The b-wave amplitude was measured at a flash intensity of 6.325 e-3   cd*s/m^2^. The average amplitude of b-waves was used as a major parameter to reflect retinal function.

### In-situ detection of cell death in the retina by the TUNEL assay

To detect apoptotic cells, retinal sections were processed for transferase dUTP nick-end labeling (TUNEL) using a kit (Roche) according to the manufacturer’s instructions.

### Statistical analysis

All data were analyzed with SPSS version 17 (IBM) and are presented as the mean ± SD. Multiple comparisons were performed by one-way analysis of variance (ANOVA), followed by Bonferroni post-hoc tests. Statistical significance was designated as *p* < 0.05. Graphing was performed using GraphPad Prism 5 software.

## Results

### In-vitro characterization of hPDLSCs

**Fig. 1:**
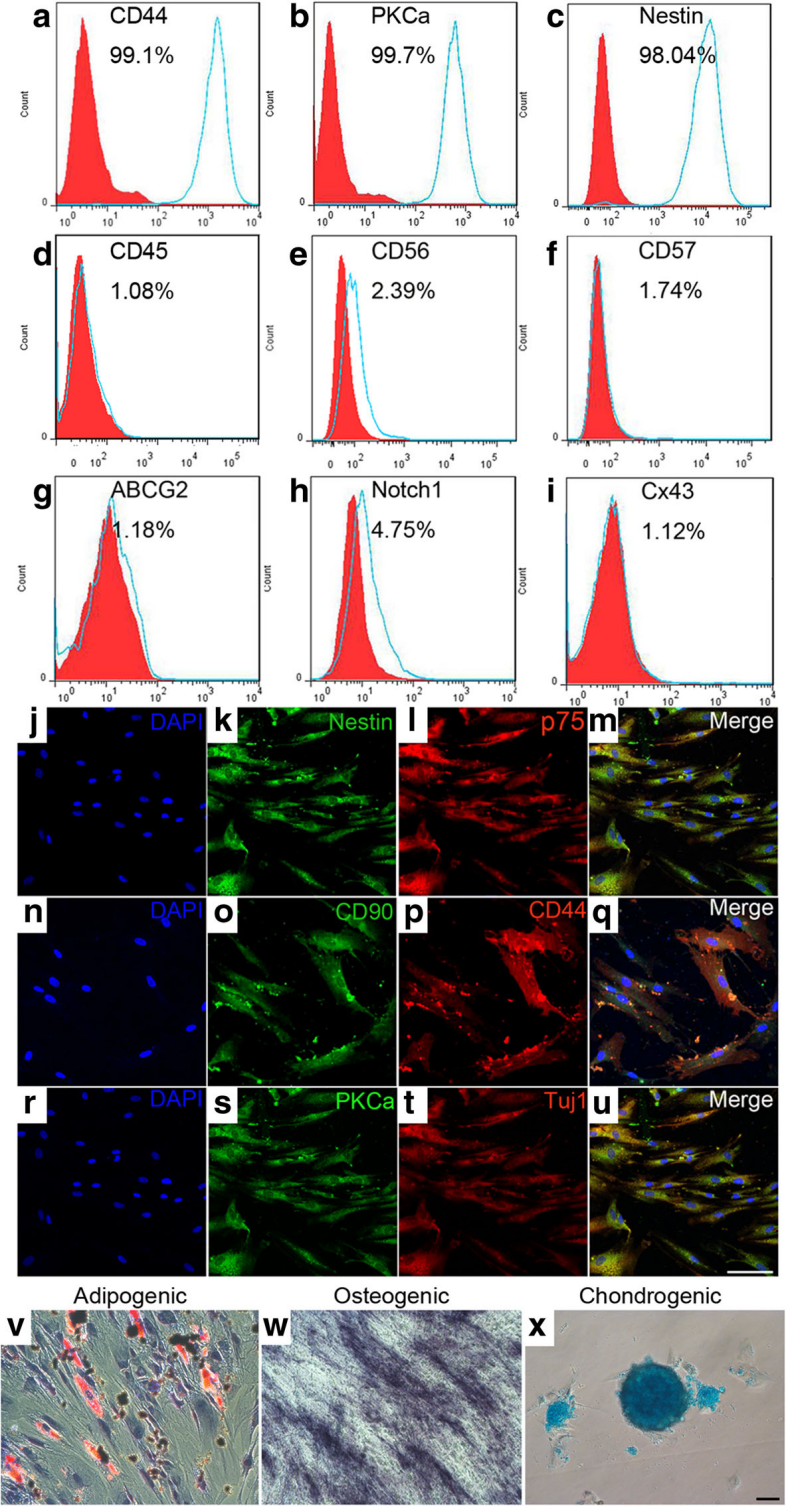
Characterization of hPDLSCs. **a–i** Flow cytometry analysis showing that hPDLSCs express CD44 (**a**), PKCα (**b**), and Nestin (**c**) and are negative for CD45 (**d**), CD56 (**e**), CD57 (**f**), ABCG2 (**g**), Notch1 (**h**), and Cx43 (**i**). Open histograms indicate specific antibodies, and filled histograms represent isotype controls. **j–u** Representative images for immunocytochemical analysis of hPDLSCs. hPDLSCs express Nestin (**k**), p75 (**l**), CD90 (**o**), CD44 (**p**), PKCα (**s**), and Tuj1 (**t**). Cell nuclei detected with DAPI (**j**, **n**, **r**), and merged images (**m**, **q**, **u**). Scale bar = 100 μm. **v–x** Trilineage differentiation of hPDLSCs. Oil Red O staining (**v**), alkaline phosphatase activity assay (**w**), and Alcian Blue staining (**x**) of hPDLSCs after 2 weeks of adipogenic, osteogenic, and chondrogenic induction, respectively. Scale bar = 200 μm

Isolated hPDLSCs were characterized prior to utilization. Immunocytochemistry evaluation demonstrated that hPDLSCs stained positive for neural crest stem cell markers (Nestin and p75) and early neuronal marker Tuj1 (Fig. 1k, l, t). Interestingly, a putative rod bipolar cell marker, PKCα, was also expressed extensively by hPDLSCs (Fig. 1s). hPDLSCs also abundantly expressed typical MSC markers (CD44 and CD90) (Fig. 1o, p). Corroborating these results, flow cytometry analysis revealed high expression of CD44 (99.1%), PKCa (99.7%), and Nestin (98.04%) in human PDLSC isolates, while CD45, CD56, CD57, ABCG2, Notch1, and Connexin43 (Cx43) were absent or expressed at low levels (<5%) (Fig. 1a–i).

### Multilineage differentiation capabilities of hPDLSCs

The multipotency of hPDLSCs was determined through evaluation of the adipogenic, osteogenic, and chondrogenic differentiation capacities. After 2 weeks of cultivation in adipogenic medium, the intracellular lipid accumulation in hPDLSCs was observed by Oil Red O staining (Fig. 1v). hPDLSCs exhibited the capacity to differentiate into the odontogenic lineage, as illustrated by ALP staining, following 2 weeks of induction in osteogenic medium, and the ALP-positive cells were stained blue (Fig. 1w). Moreover, chondrogenic differentiation was confirmed by positive staining for extracellular matrix proteoglycans (Fig. 1x), which are detectable in the aggregates of hPDLSC cultures, identified by Alcian blue staining.

### Survival and distribution of hPDLSCs following subretinal transplantation

**Fig. 2:**
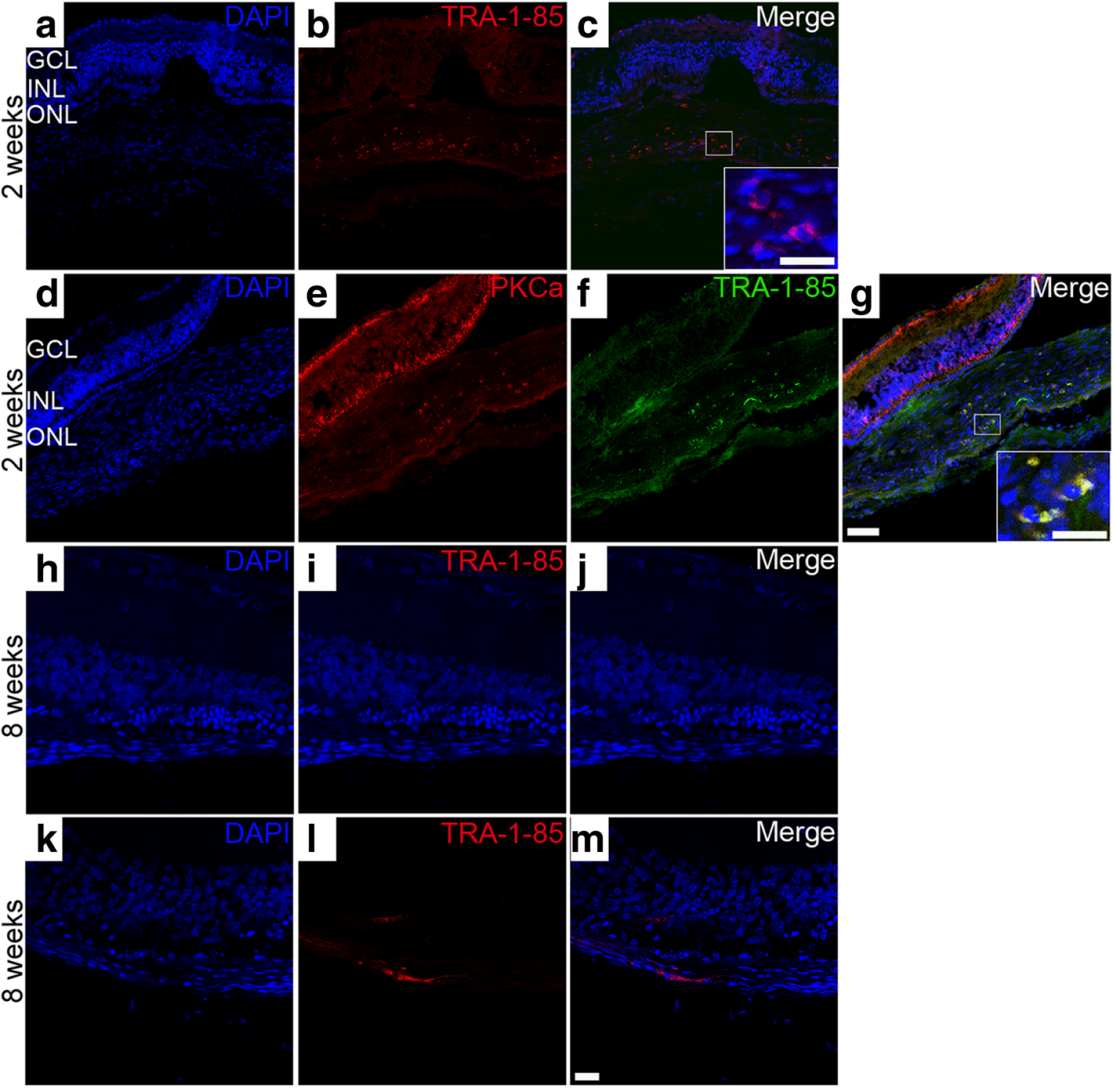
Immunohistochemical staining showing hPDLSCs in the subretinal space. Cross-sections of the eyes removed 2 weeks (**a**–**c**) and 8 weeks (**h**–**m**) post transplantation were stained for TRA-1-85. Section without primary antibody served as negative control staining (**h**–**j**). Cell nuclei counterstained with DAPI (**a**, **h**, **k**), and merged images (**c**, **j**, **m**). **d–g** hPDLSCs coexpressed PKCα (**e**) and TRA-1-85 (**f**). Cell nuclei counterstained with DAPI (**d**), and merged images (**g**). **a**–**g** Scale bar = 50 μm. **h**–**m** Scale bar = 20 μm. Inserts show higher magnification of the boxed area. Scale bars in insets = 20 μm. ONL outer nuclear layer, INL inner nuclear layer, GCL ganglion cell layer

The survival of the grafted hPDLSCs was determined by immunolabeling for human-specific marker TRA-1-85. Two weeks post transplantation, the immunohistological examination of eyes showed that TRA-1-85-immunoreactive cells were clearly detected in the subretinal space (Fig. 2a–c). The grafted hPDLSCs formed a sheet-like structure of approximately two to three layers in the subretinal space (Additional file 3: Figure S1). However, the migration behavior of the grafted cells into the host retina was seldom observed at 2 weeks post transplantation. PKCα was not only abundantly expressed in rod bipolar cells in the host retina but also colabeled with TRA-1-85 in the subretinal space (Fig. 2d–g). By 8 weeks post injection, TRA-1-85-positive cells were observed only sporadically (Fig. 2k–m) or rarely seen in retinal sections. A few grafted cells migrating into the inner retinal layer and ganglion cell layer of the host retina could be detected at 8 weeks post transplantation (arrowheads, Fig. 8). Thus, robust survival of the grafted hPDLSCs was demonstrated at 2 weeks post implantation and the number of surviving cells declined with time.

### Human PDLSC transplants preserve retinal function

**Fig. 3:**
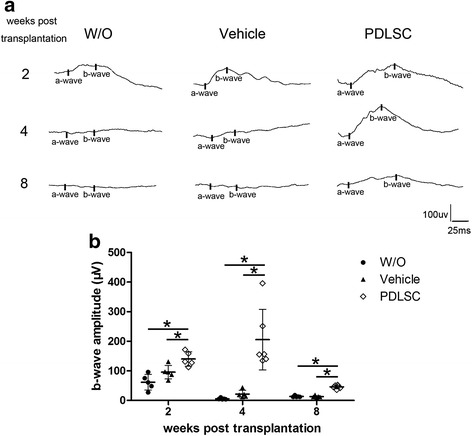
Human PDLSC transplantation enhanced ERG b-wave amplitude. **a** Representative scotopic ERG recordings from untreated RCS rats (W/O) and RCS rats injected with PBS (vehicle) or hPDLSCs at 2, 4, and 8 weeks post transplantation. **b** The b-wave response amplitudes of the PDLSC-injected group were significantly higher than those of the untreated or PBS-injected groups at 2, 4, and 8 weeks post transplantation. *n ≥* 4 eyes per group at each time point. *p < 0.05. PDLSC periodontal ligament-derived stem cell

To evaluate the functional improvement by hPDLSC transplantation, ERG testing was performed in animals that received hPDLSC treatment or PBS (vehicle) or were left untreated (W/O). Representative ERG traces recorded in scotopic conditions at 2, 4, and 8 weeks post implantation under dark conditions for each experimental group are shown in Fig. 3a. The ERG b-wave amplitudes of the untreated (W/O) animals markedly decreased and exhibited barely detectable responses at 7 weeks postnatal and thereafter. The functional improvement of the ERG signal was detected by hPDLSC transplantation at 2 weeks post engraftment, as shown by the significantly higher b-wave amplitudes (143.41 ± 24.35 μV) compared with both untreated (61.63 ± 30.17 μV) and PBS-treated (95.22 ± 32.38 μV) controls (*p* < 0.05) (Fig. 3b). Specifically, substantial ERG responses were detected with a b-wave amplitude of 204.90 ± 104.26 μV in eyes receiving hPDLSC treatment, while b-wave signals were nearly unrecordable at 4 weeks post implantation in nontreated (13.93 ± 2.98 μV) and PBS-treated (29.44 ± 14.87 μV) eyes (Fig. 3b). Although the ERG b-wave amplitudes were attenuated (dropped to 45.89 ± 6.45 μV) at 8 weeks following human PDLSC engraftment, the preservation of retinal function was still observed, as the mean b-wave amplitudes were still significantly higher than those observed either in untreated (12.57 ± 2.84 μV) or PBS-treated (13.01 ± 3.69 μV) eyes (*p* < 0.05) (Fig. 3b). There were no significant differences in the ERG b-wave amplitudes recorded in untreated or PBS-treated eyes at all time points examined. These results demonstrated that subretinal transplantation of hPDLSCs in RCS rats could provide significant functional benefit and the functional improvements could be detected up to 8 weeks after transplantation.

### Human PDLSC transplants ameliorate photoreceptor degeneration and preserve photoreceptor cell structure

**Fig. 4:**
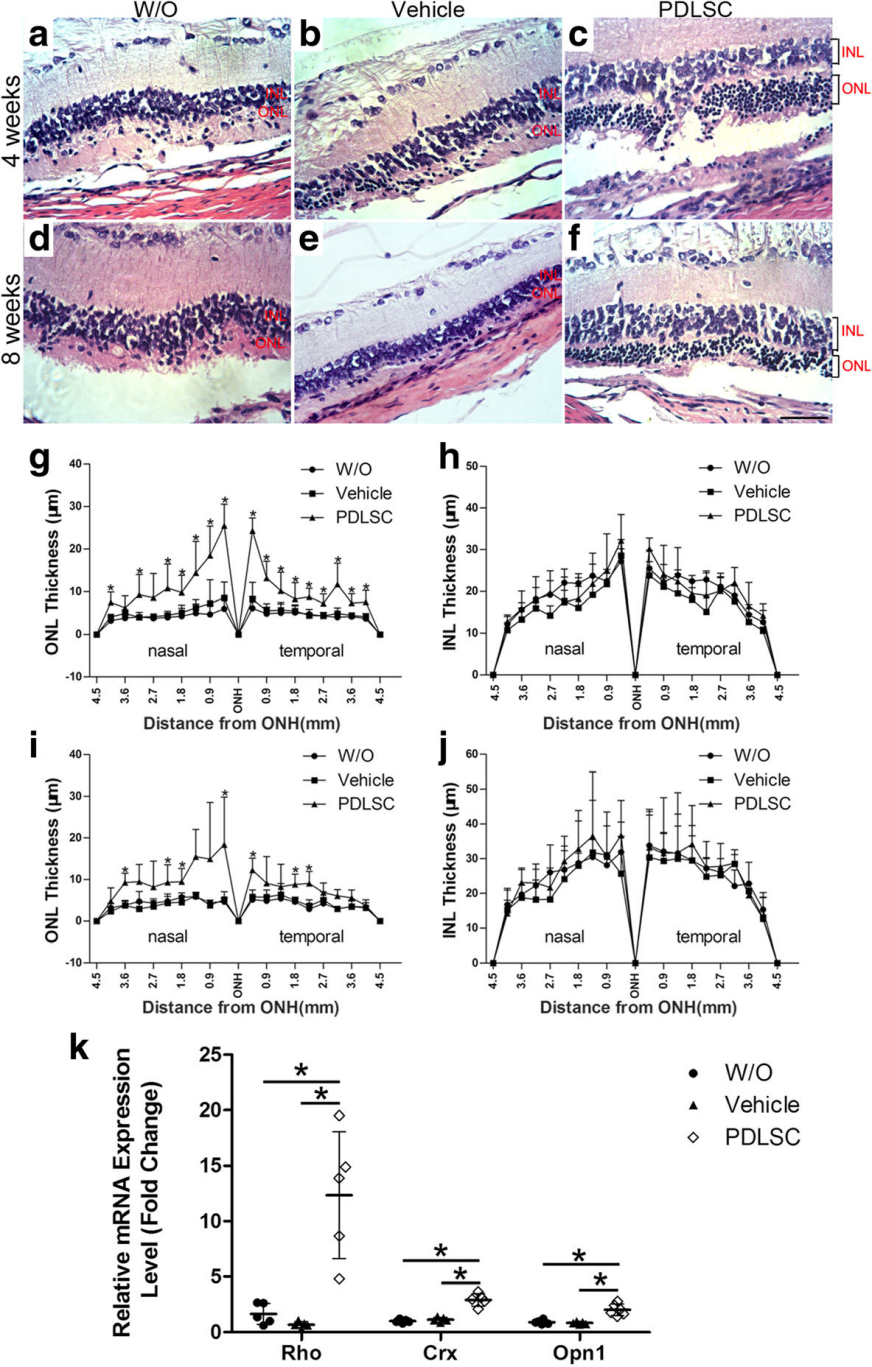
Human PDLSC transplantation preserved photoreceptors. **a–f** H&E-stained retinal sections showed the marked preservation of the ONL in human PDLSC-treated eyes (**c**, **f**) compared with untreated (W/O, **a**, **d**) and PBS-treated (vehicle, **b**, **e**) eyes at 4 weeks (**a**–**c**) and 8 weeks (**d**–**f**) post transplantation. Scale bar = 50 μm. **g–j** ONL and INL thickness of untreated RCS rats and rats receiving human PBS (vehicle) or PDLSC treatment at 4 weeks (**g**, **h**) and 8 weeks (**i**, **j**) post transplantation. **k** Real-time RT-PCR analysis showing expression levels of Rho, Crx, and Opsin in human PDLSC-treated retinas were significantly higher than those in untreated and PBS-treated eyes at 4 weeks post transplantation. Data presented as mean ± SD. *n ≥* 4 retinas per group at each time point. *p < 0.05. ONL outer nuclear layer, INL inner nuclear layer, PDLSC periodontal ligament-derived stem cell

**Fig. 5:**
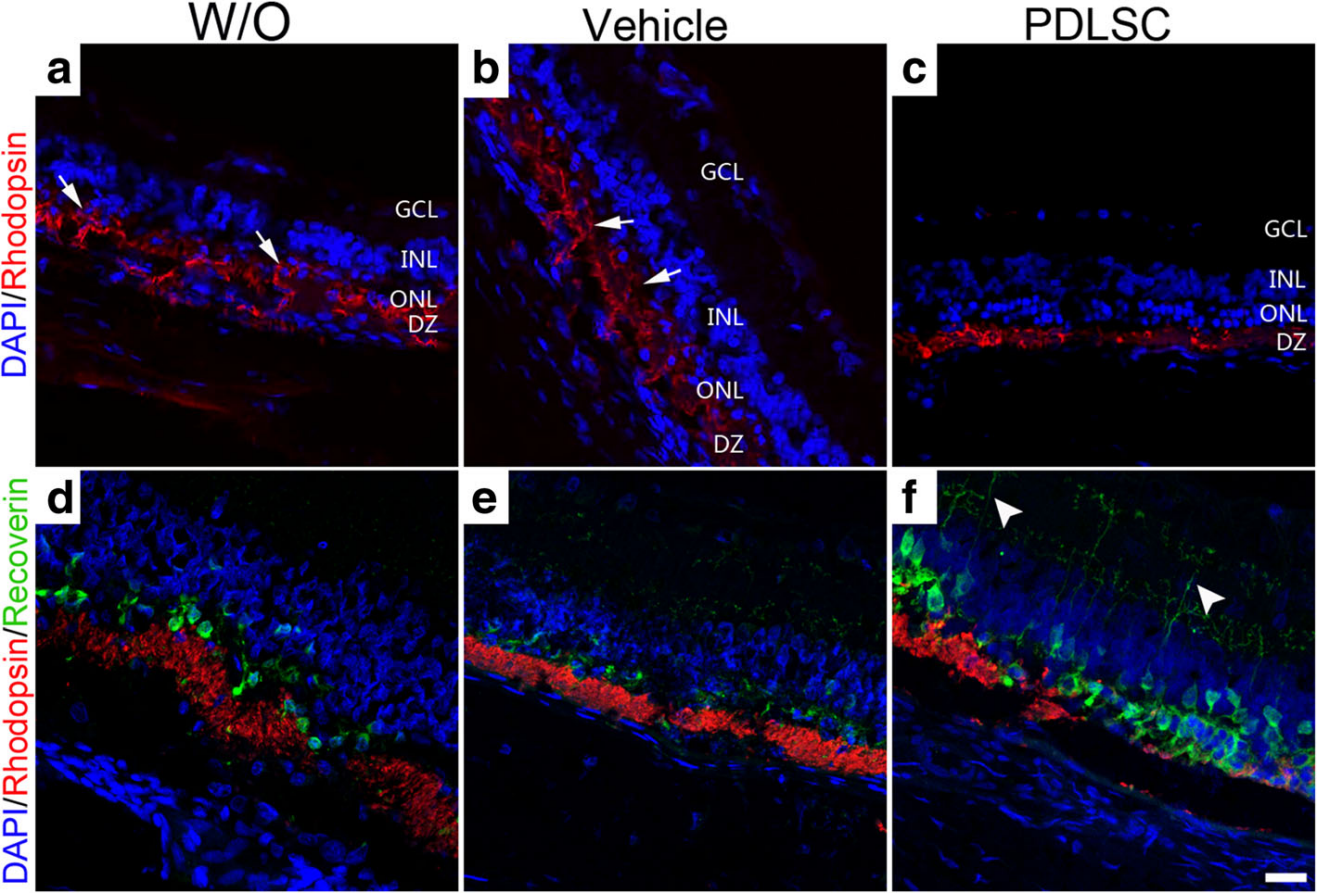
Subretinal transplantation of hPDLSCs preserved photoreceptor cell structure. **a**–**c** Confocal images of immunolabeled retinal sections showing rhodopsin staining (red) with DAPI counterstaining (blue) in untreated retinas (W/O, **a**) and retinas injected with PBS (vehicle, **b**) or hPDLSCs (PDLSC, **c**) at 4 weeks post transplantation. Arrows indicate aberrant accumulation of rhodopsin in rod cell bodies. **d**–**f** Images of retinas from untreated, PBS-treated, and PDLSC-treated RCS rats immunostained for rhodopsin (red) and recoverin (green) with DAPI counterstaining (blue). Anti-recoverin-labeled photoreceptor synaptic terminals were seen in human PDLSC-treated retinal sections at 4 weeks post transplantation (arrowheads). Scale bar = 20 μm. DZ debris zone, ONL outer nuclear layer, INL inner nuclear layer, GCL ganglion cell layer, DAPI 4′,6′-diamidino-2-phenylindole, PDLSC periodontal ligament-derived stem cell

**Fig. 6:**
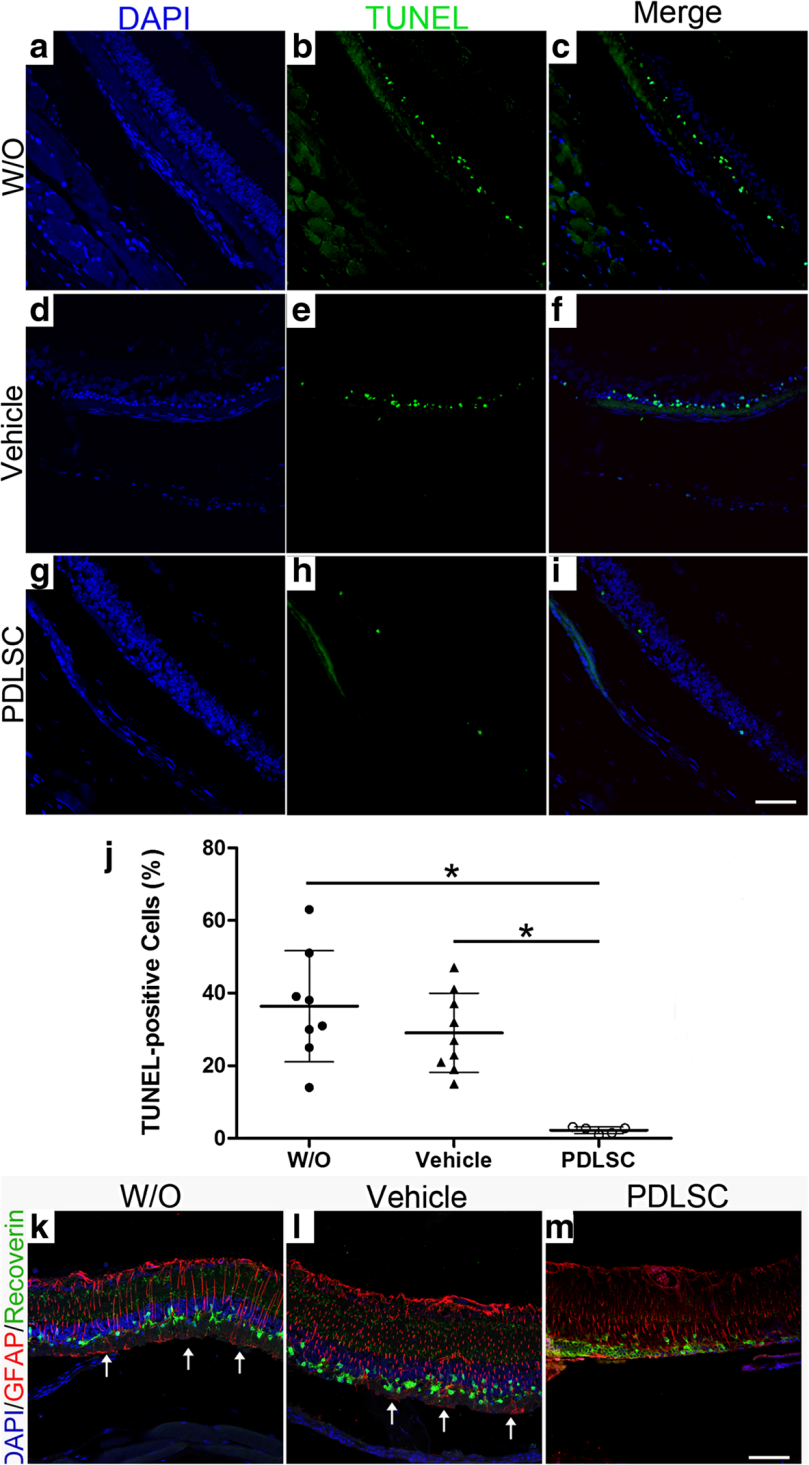
Subretinal transplantation of hPDLSCs reduced apoptosis and gliosis of rat retinas. **a**–**i** Representative images showing TUNEL labeling of apoptotic ONL cells (**b**, **e**, **h**) with DAPI counterstaining (**a**, **d**, **g**) and merged images (**c**, **f**, **i**). Scale bar = 50 μm. **j** Quantitative analysis showing percentage of TUNEL-positive nuclei was significantly smaller in human PDLSC-treated eyes than in the untreated (W/O) or PBS-treated (vehicle) eyes at 4 weeks post transplantation. *n ≥* 4 eyes per group. *p < 0.05. **k**–**m** GFAP immunoreactivity in untreated (**k**), PBS-treated (**l**), and human PDLSC-injected (**m**) retinas. GFAP-labeled Müller cell processes (upward-pointing arrows in **k** and **l**) extended into the subretinal space. Scale bar = 50 μm. DAPI 4′,6′-diamidino-2-phenylindole, PDLSC periodontal ligament-derived stem cell, TUNEL terminal deoxynucleotidyl transferase-mediated dUTP nick-end labeling

Retinal degeneration in RCS rats is characterized by the loss of photoreceptor cell bodies in the ONL and the thinning of this layer. Retinal sections were analyzed for their ONL thickness to assess whether hPDLSC grafts could preserve photoreceptors. Representative rat retinal histology images from different experimental groups at 4 weeks (Fig. 4a–c) and 8 weeks (Fig. 4d–f) post engraftment are shown. The remarkable protection of photoreceptors in hPDLSC-grafted retinas was observed with an ONL thickness of 7–10 nuclei at 4 weeks post engraftment (Fig. 4c) and 3–6 nuclei at 8 weeks post engraftment (Fig. 4f). This was in marked contrast to the ONL with just one discontinuous row of photoreceptors in either the PBS-treated or untreated RCS retinas (Fig. 4a, b, d, e). Quantitative analysis revealed that the ONL thickness of the eyes receiving hPDLSCs was significantly higher than that of the untreated eyes and eyes injected with PBS at 4 weeks (Fig. 4g, *p* < 0.05) and 8 weeks (Fig. 4i, *p* < 0.05) post engraftment. Moreover, the structure was rescued by hPDLSC transplantation, which was observed along most of the retina. No significant difference in the INL thickness was observed between the three groups at either 4 weeks (Fig. 4h) or 8 weeks (Fig. 4j) post engraftment. Furthermore, real-time PCR analysis was performed to assess the rescue effect of photoreceptor transcripts by the subretinal transplantation of hPDLSCs at 4 weeks post engraftment. Figure 4k demonstrates the significantly higher expression levels of photoreceptor-specific transcripts, including Rho, Crx, and Opsin, in the retinas from hPDLSC-injected eyes compared with those from PBS-injected or nontreated eyes. Thus, transplanted hPDLSCs resulted in the preservation of the photoreceptor layer and retinal histology in RCS rats.

The deleteriousc-mer proto-oncogene tyrosine kinase (Mertk) mutation in RCS rats disrupts RPE phagocytosis, which results in the accumulation of undigested photoreceptor outer segments (POS), termed debris zones (DZ), which ultimately compromise vision [21]. In untreated (Fig. 5a) or PBS-treated (Fig. 5b) retinas, the rhodopsin-stained photoreceptor outer segment layer is reduced to a debris zone in the subretinal space, and a strong aberrant accumulation of rhodopsin staining in rod cell bodies was observed. In contrast to the control retinas, rhodopsin-stained photoreceptor cell debris was less prominent (Fig. 5c). Additionally, there was no aberrant rhodopsin staining in rod cell bodies, which was clearly identified in untreated (arrows in Fig. 5a) and PBS-treated (arrows in Fig. 5b) retinas.

In sections of untreated (Fig. 5d) and PBS-treated (Fig. 5e) retinas at 4 weeks post transplantation that were double-stained for rhodopsin and recoverin, the photoreceptors exhibited disorganized cell polarity and lacked the distinct synaptic terminals. However, photoreceptor cell polarity was maintained, and the recoverin-immunoreactive photoreceptor synaptic terminals were prominently labeled (arrowheads) in hPDLSC-grafted retinas (Fig. 5f).

Collectively, these data suggested that hPDLSC transplantation was efficient not only in slowing down photoreceptor cell loss, but also in preserving the existing photoreceptor cell structure, therefore demonstrating its potential utility in photoreceptor protection and therapeutic effect in RCS rats.

### Human PDLSC transplants protect photoreceptors against apoptosis and gliosis

Apoptosis is the dominant mechanism of photoreceptor degeneration in RCS rats [22]. To determine the capacity of hPDLSC transplants in delaying the activation of apoptotic photoreceptor cell death in RCS rats, TUNEL staining was performed on retinal cryo-sections at 4 weeks after engraftment. Numerous TUNEL-positive cells were observed in the ONL, indicative of abundant photoreceptor apoptosis in untreated (Fig. 6a–c) and PBS-treated (Fig. 6d–f) eyes and confirming the apoptotic cell death in this model. In contrast, TUNEL-positive nuclei were rarely detectable in hPDLSC-treated eyes (Fig. 6g–i). Quantitative analysis showed that the hPDLSC transplants significantly reduced the percentage of TUNEL-positive cells compared with that in the untreated or PBS-treated groups (Fig. 6j, *p <* 0.05).

By immunostaining retinas at 4 weeks post grafting, we studied the expression levels and distribution of GFAP, an indicator of Müller glia reactivity in degenerating retinas. Strong GFAP staining was observed in the retinas from untreated (Fig. 6k) and PBS-treated (Fig. 6l) eyes. In addition, the GFAP-labeled Müller cell processes were observed occasionally, extending beyond the outer limiting membrane (OLM) and sprouting into the subretinal space (arrows in Fig. 6k, l). In contrast, in hPDLSC-treated eyes the intensity of GFAP immunoreactivity was reduced, and no staining was detected in the subretinal space (Fig. 6m), indicating protection of rat photoreceptors from glial stress.

### hPDLSCs express neurotrophic factors in vitro

**Fig. 7:**
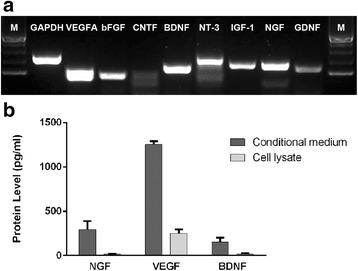
Neurotrophic factor expression in hPDLSCs. **a** RT-PCR analysis of various neurotrophic factor transcripts in hPDLSCs. GAPDH used as the PCR control. M marker (Takara 100 bp DNA Ladder). **b** ELISA NGF, VEGF, and BDNF protein levels in both supernatants and cell lysates of hPDLSCs. *n* = 4 independent experiments. BDNF brain-derived neurotrophic factor, bFGF bioactive basic fibroblast growth factor, CNTF ciliary neurotrophic factor, GDNF glial cell line-derived neurotrophic factor, IGF-1 insulin-like growth factor 1, NGF nerve growth factor, NT-3 neurotrophin-3, VEGF vascular endothelial growth factor

Various studies have hypothesized that MSCs primarily exert their therapeutic benefits on retinal repair by paracrine action [11, 23, 24]. We next examined the mechanism underlying the morphological and functional recovery following hPDLSC transplantation and tested the potency of hPDLSCs in secreting trophic factors, which have been implicated previously in neuronal survival. Semiquantitative RT-PCR analysis showed that hPDLSCs expressed the mRNA transcripts for vascular endothelial growth factor A (VEGFA), bioactive basic fibroblast growth factor (bFGF), BDNF, neurotrophin-3 (NT-3), insulin-like growth factor 1 (IGF-1), NGF, and glial cell line-derived neurotrophic factor (GDNF). However, the mRNA expression of ciliary neurotrophic factor (CNTF) was not detected in hPDLSCs (Fig. 7a). The ELISA data confirmed the prominent expression of NGF (296.67 ± 92.92 pg/ml), VEGF (1255.14 ± 35.70 pg/ml), and BDNF (153.33 ± 49.33 pg/ml) in the supernatant of hPDLSC culture (Fig. 7b).

## Discussion

This study demonstrates for the first time that hPDLSCs provide a significant therapeutic benefit in sustaining retinal function for up to 8 weeks and delay photoreceptor degeneration along most of the retina after implantation into the subretinal space of RCS rats. Our data suggest that hPDLSCs may represent a promising therapeutic alternative for RD.

hPDLSCs are thought to originate from the cranial neural crest. They abundantly express MSC markers and exhibit the unique features of neuroectodermal stem cells [25, 26]. Consistently, our immunocytochemical staining and flow cytometry analysis revealed that hPDLSCs presented a high rate of positivity (> 90%) for conventional cell surface markers of MSCs, including CD44 and CD90, indicating their mesenchymal characteristics. A considerable amount of hPDLSCs express neural cell markers, including PKCα (99.7%) and Nestin (98.04%), which may be related to the neural crest origin of PDL. Our study also provides evidence that hPDLSCs represent a population of postnatal stem cells capable of multilineage differentiation into adipogenic, osteogenic, and chondrogenic lineages when grown in their respective induction media.

ERG recordings at 2, 4, and 8 weeks post transplantation showed substantial preservation with hPDLSC treatment compared with that of controls, indicating the therapeutic efficacy of hPDLSC transplantation. However, the b-wave amplitudes decreased between 4 and 8 weeks post treatment, suggesting that the degeneration process was delayed rather than halted by the cell treatment. We proposed that poor cell survival may be an important factor influencing the therapeutic efficacy of hPDLSC treatment following transplantation. Immunohistochemical staining with human specific marker TRA-1-85 revealed a substantial number of transplanted hPDLSCs in the subretinal space at 2 weeks post transplantation. However, the number of transplanted cells dramatically declined with time, with a negligible amount of cells remaining in the host retina at 4 weeks post transplantation. Previous studies have reported a similar fallout of donor cells with time and limitations of the therapeutic effects using subretinal transplants of human MSCs [4, 27], NSCs [4], ADSCs [23], and human umbilical cord tissue-derived stem cells (UTCs) [28] in retinal degeneration models. One likely explanation is that transplanted cell survival may have been compromised because of the hostile environment of the diseased retina. Immune-mediated xenograft rejection may also pose a major obstacle to the survival duration of graft cells. Whether graft cell survival and therapeutic effects could be improved by utilizing a bioscaffold or a second transplantation warrants further study. It is also conceivable that autologous hPDLSC transplantation may have the advantage of reducing the risk of rejection and present prolonged therapeutic benefits for the treatment of retinal degeneration.

It is now widely recognized that adult stem cells exert a beneficial influence on retinal repair predominantly by paracrine action through the secretion of trophic and anti-inflammatory factors [27, 29, 30]. Like other MSCs, the extensive neurotrophic secretome of DPSCs has been documented widely [11, 31, 32]. Furthermore, DPSCs were found to secrete VEGF, NGF, BDNF, and NT-3 at higher concentrations than BMSCs [11, 33]. Mead et al. [11, 12] reported a more pronounced paracrine-mediated RGC survival and neurite outgrowth by transplanted human DPSCs compared with these variables after BMSC transplantation in animal models of optic nerve injury and glaucoma. In contrast to DPSCs, few data exist to date describing the neurotrophic factor secretion and neuroregenerative/neuroprotective activities of hPDLSCs. Here, we report for the first time the expression by hPDLSCs of a variety of neurotrophic factors, including VEGFA, bFGF, BDNF, NT-3, IGF-1, NGF, and GDNF, as determined by PCR and ELISA measurements. In the eye, VEGF, NGF, bFGF, BDNF, NT-3, IGF-1, and GDNF are widely considered neuroprotective, possibly by an anti-apoptotic mechanism, for photoreceptor survival [23, 34–39]. We reported recently that hADSCs preserved retinal structure and visual function in RCS rats, at least in part by secreting VEGF [23]. In this study, similar quantities of VEGF expression were detected in hPDLSCs (1255.14 ± 35.70 pg/ml) and hADSCs (1268.72 ± 334.67 pg/ml). In this scenario, hPDLSCs may also serve as a source for supply of these neurotrophic and neuroprotective factors to the diseased retina for photoreceptor survival and therefore delay retinal degeneration in RCS rats.

Apoptotic cell death has been shown to be the common endpoint of photoreceptors in most RD [40]. Undigested POS accumulates as toxic debris in the subretinal space of RCS rats, which instigates photoreceptor apoptosis [21]. Our data showed slowed retinal pathogenesis in RCS rats with an observed reduction in the photoreceptor debris zone and the TUNEL-positive ONL cells in the hPDLSC transplantation group compared with those in the PBS group and the untreated group. This result was consistent with the findings of Tzameret et al. [41], which indicated reduced DZ and retinal structure improvement by human BMSC transplantation. These findings further corroborate the hypothesis that paracrine-mediated anti-apoptotic effects could be partially responsible for the beneficial effects of hPDLSCs on the degenerating retina.

Histological analysis and ERG recordings demonstrated the obvious delay of photoreceptor degeneration and maintenance of retinal function that persisted up to 8 weeks post transplantation. In contrast, there were few hPDLSCs remaining in the retina. Our findings support the notion that achieving a therapeutic benefit does not require the presence or long-term survival of engraftment, leading to the hypothesis that trophic factors secreted by transplanted hPDLSCs during the first 2 weeks may be sufficient to delay retinal degeneration for a longer duration.

**Fig. 8:**
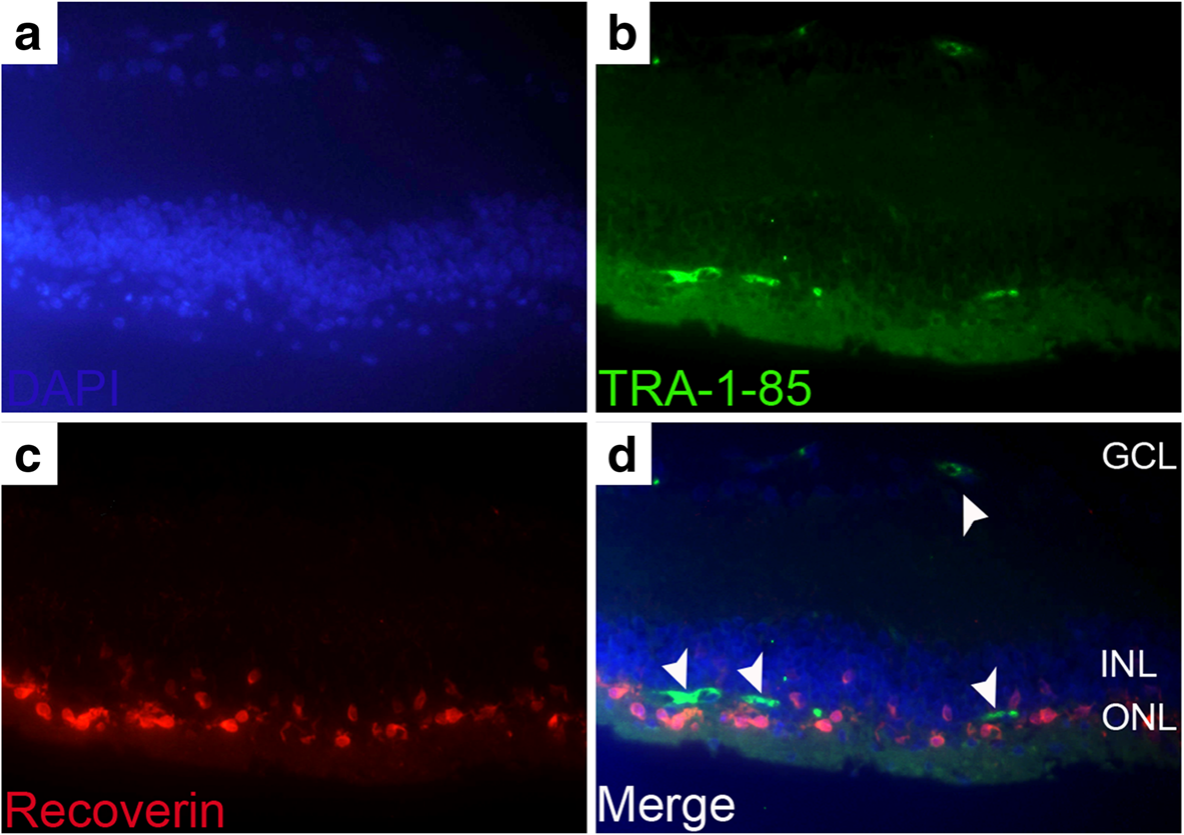
Fluorescence microscope images of retinal sections injected with hPDLSCs at 8 weeks post transplantation. Retinal sections were double-stained with human antigen TRA-1-85 (**b**) and recoverin (**c**), with DAPI counterstaining (**a**) which showed no colocalization (**d**). Arrowheads indicate migrating hPDLSCs. ONL outer nuclear layer, INL inner nuclear layer, GCL ganglion cell layer

Because hPDLSCs constitutively express PKCα and Tuj1, even in an undifferentiated state, the differentiation of these cells into bipolar cells or RGCs after engraftment is contentious. To determine whether the grafted hPDLSCs have the potential to differentiate into photoreceptor cells in the retina, double labeling of the grafted cells, as indicated by TRA-1-85-positive staining with recoverin, was performed. Our data showed evidence of a few grafted hPDLSCs migrating into the host retina but failed to express recoverin at 8 weeks post transplantation (Fig. 8). The disparity between the reported successful photoreceptor differentiation of hPDLSCs in vitro in our previous study [18] and the lack of differentiation in vivo could be due in part to the pathological microenvironment of the diseased retina, which presents a vastly different environment from the carefully controlled in-vitro setting. Another possible explanation is that the retinal neuronal differentiation of hPDLSCs may take a much longer time to develop. The poor survival of hPDLSCs after engraftment may diminish their chance for subsequent differentiation. Further studies confirming the neuronal differentiation potential of hPDLSCs by developing methods to improve graft cell survival have not yet been conducted.

To the best of our knowledge, this is the first demonstration of the preservation of photoreceptors and the maintenance of retinal function by the subretinal transplantation of hPDLSCs. Here, we found no evidence of untoward pathological manifestations or tumor-like transformations up to at least 8 weeks after transplantation during the experimental period. The advantages of being an ethical cell source, being relatively easy to access from teeth, having low immunogenicity, and having the potential for an off-the-shelf product without a predifferentiation process before transplantation, along with the nontumorigenic and distinctive trophic characteristics, make autologous and allogeneic hPDLSC transplantation a promising paracrine-mediated therapy for retinal degenerative diseases.

In summary, this study provides a proof of principle for the use of hPDLSCs to treat retinal degeneration. For translational implications, it remains critical to investigate the cells’ long-term safety and optimize strategies to enhance the therapeutic benefits of hPDLSC transplantation in future work.

## Conclusions

For the first time, this study demonstrates the potential of neural crest-originated hPDLSCs to act as a cellular therapy for retinal degeneration by significantly preserving retinal structure and retinal function in RCS rats. The underlying mechanisms may have been attributed to the paracrine-mediated protective effects by human PDLSC transplantation. Collectively, the robust retinal histological and functional improvements by human PDLSC transplantation presented here advocate further exploration of these cells for use in treating retinal degenerative diseases such as RP and AMD.

## Additional files

Additional file 1: Table S1. Presenting primary antibodies used for immunocytochemistry and flow cytometry (DOCX 60 kb)

Additional file 2: Table S2. Presenting gene expression primers (DOCX 61 kb)

Additional file 3: Figure S1. Showing grafted hPDLSC distribution at 2 weeks after transplantation. Immunohistochemical staining showing PKCα-positive hPDLSCs were sheetlike (**b**) and spread in the subretinal space (outlined area in **c**). Cell nuclei counterstained with DAPI (**a**) (TIF 1458 kb)

## Abbreviations

ADSC: Adipose-derived stem cell
AMD: Age-related macular degeneration
BDNF: Brain-derived neurotrophic factor
bFGF: Bioactive basic fibroblast growth factor
BMSC: Bone marrow mesenchymal stem cell
CNTF: Ciliary neurotrophic factor
DAPI: 4′,6′-diamidino-2-phenylindole
DFPC: Dental follicle progenitor cell
DPSC: Dental pulp stem cell
ELISA: Enzyme-linked immunosorbent assay
ERG: Electroretinography
ESC: Embryonic stem cell
GDNF: Glial cell line-derived neurotrophic factor
hPDLSC: Human periodontal ligament-derived stem cell
IGF-1: Insulin-like growth factor 1
iPSC: Induced pluripotent stem cell
MSC: Mesenchymal stem cell
NGF: Nerve growth factor
NSC: Neural stem cell
NT-3: Neurotrophin-3
ONL: Outer nuclear layer
PBS: Phosphate-buffered saline
RCS: Royal College Surgeons
RD: Retinal degeneration
RGC: Retinal ganglion cell
RP: Retinitis pigmentosa
RPE: Retinal pigmented epithelial
SCAP: Stem cells from apical papilla
SHED: Stem cells from human exfoliated deciduous teeth
TUNEL: Terminal deoxynucleotidyl transferase-mediated dUTP nick-end labeling
UTC: Umbilical cord tissue-derived stem cell
VEGF: Vascular endothelial growth factor
VEGFA: Vascular endothelial growth factor A
