# Supplementary material for: Adult human periodontal ligament-derived stem cells delay retinal degeneration and maintain retinal function in RCS rats
Source: Stem Cell Res Ther. 2017 Dec 22;8:290. doi: 10.1186/s13287-017-0731-y (PMC5741902; doi:10.1186/s13287-017-0731-y)
Supplement: Supplementary file 1 — Presenting primary antibodies used for immunocytochemistry and flow cytometry (DOCX 60 kb) [file 13287_2017_731_MOESM1_ESM.docx]

| **Antibody** | **Type** | **Source** | **Dilution** |
| --- | --- | --- | --- |
| Nestin | Mouse monoclonal | Millipore | 1:200 |
| Tuj1 | Mouse monoclonal | Promega | 1:100 |
| p75 | Rabbit polyclonal | Abcam | 1:100 |
| CD44 | Rabbit polyclonal | Abcam | 1:100 |
| PE conjugated mouse anti-human CD44 |  | BD Pharmingen | 5 μl per Test |
| CD90 | Mouse moloclonal | Abcam | 1:100 |
| FITC conjugated mouse anti-human CD56 |  | BD Pharmingen | 5 μl per Test |
| FITC conjugated Mouse anti-human CD57 |  | BD Pharmingen | 20 μl per Test |
| PE conjugated Mouse anti-human ABCG2 |  | BD Pharmingen | 5 μl per Test |
| PE mouse anti-human CD45 |  | BD Pharmingen | 20 μl per Test |
| Notch1 | Rabbit moloclonal | Abcam | 1:100 |
| APC conjugated Mouse anti-human Cx43 |  | R & D Systems | 5 μl per Test |
| Tra-1-85 | Mouse monoclonal | R & D Systems | 1:200 |
| GFAP | Mouse monoclonal | Sigma | 1:400 |
| Rhodopsin | Mouse moloclonal | Thermo | 1:100 |
| PKCα | Rabbit moloclonal | Abcam | 1:250 |
| Recoverin | Rabbit polyclonal | Proteintech | 1:200 |
