# Supplementary material for: Adult human periodontal ligament-derived stem cells delay retinal degeneration and maintain retinal function in RCS rats
Source: Stem Cell Res Ther. 2017 Dec 22;8:290. doi: 10.1186/s13287-017-0731-y (PMC5741902; doi:10.1186/s13287-017-0731-y)
Supplement: Supplementary file 2 — Presenting gene expression primers (DOCX 61 kb) [file 13287_2017_731_MOESM2_ESM.docx]

| **Gene amplified** | **Forward** | **Reverse** | **Size (bp)** |
| --- | --- | --- | --- |
| *GAPDH* | GAA GGT GAA GGT CGG AGT | GAA GAT GGT GAT GGG ATT TC | 226 |
| *VEGFA* | TGT TTG TAC AAG ATC CGC AGA CGT G | TCA CCG CCT CGG CTT GTC ACA TCT GCA AGT ACG TT | 119 |
| *GDNF* | TAG AAG GCT GGT GAG TGA CAA AGT A | TCA TCT AAA AAC GAC AGG TCA TCA TC | 138 |
| *bFGF* | CCG ACG GCC GAG TTG AC | TGA TAG ACA CAA CTC CTC TCT CTT CTG | 90 |
| *BDNF* | AGG GTG ATG CTC AGT AGT CAA GTG | GTT TGC AGC ATC TAG GTA ATT TTT GT | 147 |
| *CNTF* | TTA TCG TAC CTT CCA TGT TTT GTT G | GGG TAT GTA TAG CTT GAT GGA AGT CA | 98 |
| *NT-3* | TCA AAA CGG GCA ACT CTC CT | CTC GAC AAG GCA CAC ACA CA | 220 |
| *IGF-1* | CAT GTC CTC CTC GCA TCT CT | ATA CCC TGT GGG CTT GTT GA | 160 |
| *NGF* | ATA CAG GCG GAA CCA CAC TCA G | GTC CAC AGT AAT GTT GCG GGT C | 174 |
| *Rat-GAPDH* | GCC TCG TCT CAT AGA CAA GAT | TCC ACT TTG TCA CAA GAG AAG | 219 |
| *Rat-RHODOPSIN* | CGT CAC CGT ACA GCA CAA | GGA CTA CCA GGG ACC ACA | 135 |
| *Rat-OPSIN1* | GAA GGC TAC ATT GTC TCA CT | AGA AGA CGA TTC CCA CAG | 211 |
| *Rat-CRX* | GAA GAG GAA GGC AGG CAC A | GCA TAG GTC ATG GCA TAA GG | 156 |
