# Supplementary figures and images for: Adult human periodontal ligament-derived stem cells delay retinal degeneration and maintain retinal function in RCS rats
Source: Stem Cell Res Ther. 2017 Dec 22;8:290. doi: 10.1186/s13287-017-0731-y (PMC5741902; doi:10.1186/s13287-017-0731-y)

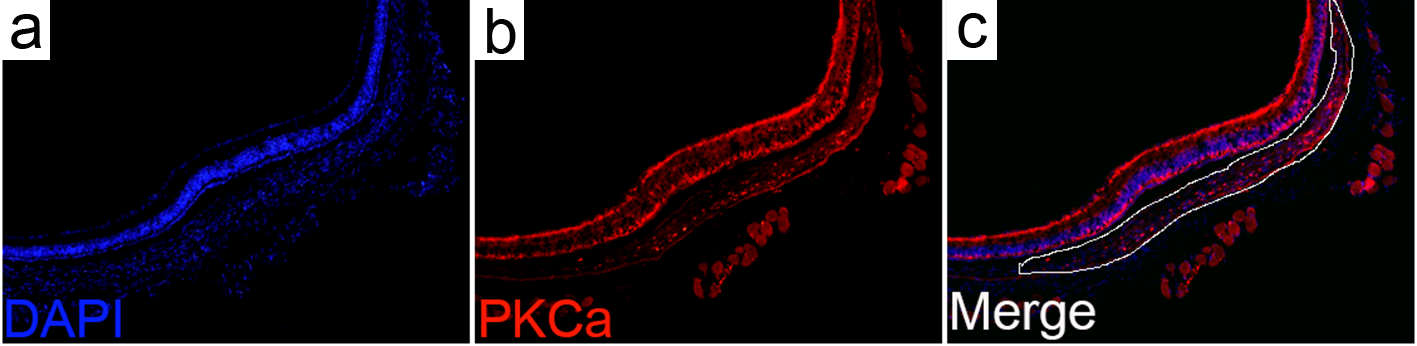

Supplement: Supplementary file 3 — Showing grafted hPDLSC distribution at 2 weeks after transplantation. Immunohistochemical staining showing PKCα-positive hPDLSCs were sheetlike (b) and spread in the subretinal space (outlined area in c). Cell nuclei counterstained with DAPI (a) (TIF 1458 kb) [file 13287_2017_731_MOESM3_ESM.tif]
